# Peritoneal dialysis-associated peritonitis caused by *Coxiella Burnetii*: A case report

**DOI:** 10.1097/MD.0000000000041407

**Published:** 2025-01-31

**Authors:** Yue Zhong, Ze-Yu Cai, Jun-Rui Wang, Qi-Ge Qi, Jian Hao

**Affiliations:** a Department of Medicine, Division of Nephrology, The Affiliated Hospital of Inner Mongolia Medical University, Hohhot, Inner Mongolia Autonomous Region, China.

**Keywords:** *Coxiella Burnetii*, PD effluent, Peritoneal dialysis-associated peritonitis, Q fever

## Abstract

**Rationale:**

Peritoneal dialysis (PD)-associated peritonitis (PDAP) is the leading cause of PD failure and discontinuation of PD. Several zoonotic pathogens could lead to the development of PDAP. *Coxiella burnetii* (*C. burnetii*) was a zoonotic pathogen and the cause of Q fever. However, reports of PDAP caused by *C. burnetii* are rare. We herein report the first case of PDAP caused by *C. burnetii* in mainland China.

**Patients concerns:**

A 45-year-old woman was admitted to our hospital with chief complaint of yellow and cloudy PD effluent for 2 days. She had undergone PD for 5 years due to end-stage renal disease. She was engaged in cattle and sheep breeding. The culture of PD effluent was negative, even for specific species, such as Mycobacteria and fungi.

**Diagnoses:**

The culture from the PD effluent tested positive for *C. burnetii* by adopting metagenomic next-generation sequencing on day 37. We diagnosed her as PDAP caused by *C. burnetii.*

**Interventions:**

Empirical treatment with multiple broad-spectrum antibiotics (including vancomycin, etimicin, piperacillin) was initially adopted. After identifying *C. burnetii* as the culprint as the PDAP, the regimen was changed to doxycycline (100 mg twice daily) and moxifloxacin (400 mg once daily) orally, leading to clinical improvement.

**Outcomes:**

The white blood cell count of the PD effluent decreased to within the normal range and the culture of PD effluent was negative for *C. burnetii* at the visit of 4 months after discharge. Also, there was no sign for recurrence.

**Lessons:**

Vigilance should be heightened for PDAP cases with negative culture of PD fluid and poor response to standard broad-spectrum antibiotic treatment, along with a history of cattle and sheep breeding. In such conditions, PD effluent should be tested to detect possible peritonitis caused by *C. burnetii,* even in patients without symptoms of fever. Prompt pathogen identification and appropriate treatment are crucial for clinical improvement of such cases.

## 1. Introduction

Peritoneal dialysis (PD) is the preferred renal replacement therapy for patients with end-stage renal disease.^[1]^ Peritoneal dialysis-associated peritonitis (PDAP) is a common and severe complication of PD, which could lead to the PD failure and the discontinuation of PD. It was reported that PDAP could be the main cause of death for at least 15% of patients undergoing PD.^[2–7]^

Gram-negative microorganisms are the second leading cause of PDAP, following gram-positive microorganisms. The most common pathogens are *Staphylococcus, Streptococcus, Enterococcus,* and *Escherichia coli*.^[8]^ Peritonitis caused by *Coxiella burnetii* (*C. burnetii*) was rarely reported, as summarized in Table 1.^[10]^ Currently, there is no established consensus regarding the management of PDAP caused by *C. burnetii*, likely due to the scarcity of reported cases. To our knowledge, this report represents the first instance of PDAP caused by *C. burnetii* documented in mainland China.

**Table 1:** List of studies on peritonitis caused by *Coxiella burnetii* (*C. burnetii*) in human across the world.

Study	Novelty	Methodology	Treatment
Chang et al^[9]^	First case report of Q fever manifested as acute peritonitis and pericarditis	Serological survey for *C. burnetii*	Two weeks of doxycyline did not resolve the fever, thus, the patient was treated with 14 days of 500 mg/day oral levofloxacin
Yilmaz et al^[10]^	First case report of Q fever manifested as peritonitis in a patient undergoing dialysis	Serological survey for *C. burnetii*	The patient received 10 days of doxycycline and ciprofloxacin, followed by 5 weeks of doxycycline, ciprofloxacin, and rifampicin

## 2. Case presentation

A 45-year-old woman with a background of hypertension was diagnosed in 2016. She had undergone PD for 5 years due to end-stage renal disease. She was admitted to our hospital with yellow and cloudy PD effluent for 2 days in October 2023. She had experienced 2 previous episodes of culture-negative PDAP. After intraperitoneal administration of vancomycin and amikacin for 15 days, the symptoms of PDAP were improved. Besides, she was involved in cattle and sheep breeding.

**Figure 1.**
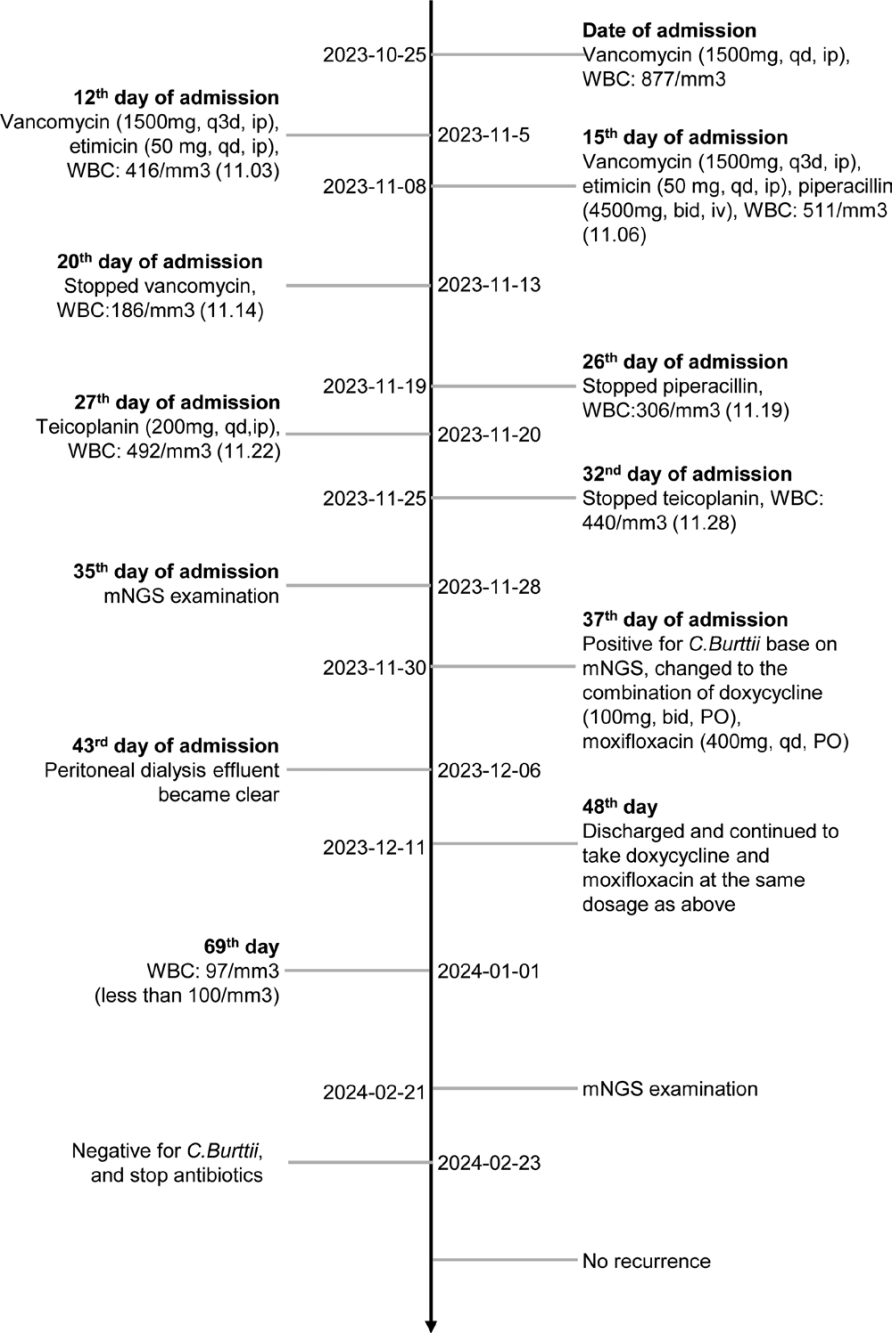
Summary of timeline. mNGS = metagenomic next-generation sequencing, WBC = white blood cell.

On the day of admission, her blood pressure was 95/65 mm Hg, pulse pressure was 88 beats/minute, and body temperature was 36.6°C. The skin at the exit-site of PD catheter was clean without any purulent discharge, indicating no evidence of exit-site infection or tunnel infection. Serum inflammatory markers showed white blood cell (WBC) count of 6.0 × 10^9^/L, neutrophils of 83.8%, C-reactive protein (CRP) level of 10.7 mg/L, N-terminal pro-B-type natriuretic peptide (NT-proBNP) level of 835 pg/mL, procalcitonin level of 0.356 ng/mL, and hemoglobin level of 105 g/L. The laboratory data from the PD effluent exhibited WBC count of 877/mm^3^ with neutrophils of 51%. Culture of PD effluent yielded negative results, including for specific species, such as Mycobacteria and fungi. Meanwhile, empirical treatment was initiated with intraperitoneal administration of vancomycin (1500 mg per day, ip). On day 2, the coronavirus disease 2019 test result was positive. By day 5, the culture of PD effluent was negative. She was informed of the potential risk of keeping the PD catheter, while she refused to remove the PD catheter. On day 12, the regimen was changed to vancomycin (1500 mg 3 times per day, ip) and etimicin (50 mg once daily, ip) with a WBC count of 416/mm^3^ and a negative culture. On day 15, the PD effluent became turbid again despite a clean exit-site of the PD catheter. The laboratory data from the PD effluent exhibited WBC count of 511/mm^3^, procalcitonin level of 0.374 ng/mL, and CRP level of 12.1 ng/ml, indicating the increased level of inflammation. Therefore, on day 15, piperacillin (4500 mg twice daily, iv) was added to the above-mentioned regimen. On day 20, vancomycin was removed from the abovementioned regimen, with a WBC count of 186/mm^3^. Notably, vancomycin was discontinued as it had been administered continuously for 2 weeks. On day 26, the piperacillin was discontinued, and the regimen was changed to etimicin (50 mg once daily, ip) with a WBC count of 306/mm^3^. On day 27, Teicoplanin (200 mg once daily, ip) was added to the regimen scheduled on day 26 with a WBC count of 492/mm^3^. On day 32, the WBC count in the PD fluid was 440/mm^3^. On day 37, the culture from the PD effluent collected on day 35 tested positive for *C. burnetii* based on metagenomic next-generation sequencing (mNGS). The regimen was therefore changed to the combination of doxycycline (100 mg twice daily, PO) and moxifloxacin (400 mg once daily, PO), which led to the rapid improvement of the symptoms. The PD effluent became clearon day 43 (6 days after the use of doxycycline and moxifloxacin). On day 48, the patient was discharged from the hospital, and she continued to adhere to the regimen for treating *C. burnetii* as previously described. Additionally, she made regular visits to the outpatient department. On day 69, the WBC count of PD effluent decreased to 97/mm^3^ with neutrophils of 80%. During the visit on February 21, 2024, the WBC count of the PD effluent decreased to within the normal range, and another sample of the PD effluent was sent for mNGS testing. After 2 days, the result of the mNGS of the PD effluent indicated that it was negative for *C. burnetii*. Consequently, doxycycline and moxifloxacin were discontinued. No recurrence was found as well. The timeline of the patients was presented in Figure 1. The informed consent was acquired from the patient.

## 3. Discussion

*C. burnetii* is a Gram-negative, intracellular microorganism, as well as a member of the genus Legionella. Therefore, it cannot be reliably observed by Gram stain.^[11]^ *C. burnetii* is highly resistant to physical and chemical factors such as ultraviolet light.^[12]^ Epidemiologic data indicated that humans are primarily infected through inhalation of infectious aerosols, either directly from the secretions (including feces, urine, after birth) of infected animals or from dust contaminated by these secretions.^[13]^ The primary hosts of *C. burnetii* are cattle, sheep, and goat.^[14]^

Notably, mNGS utilizes bioinformatics techniques to analyze microbial genomes in specific environments, providing insights into microbial diversity, community structure, evolutionary relationships, and functional predictions. In contrast to traditional microbial research methods, it enables direct analysis of microorganisms, bypassing the need for microbial culture.

Diseases caused by *C. burnetii* were first manifested as query fever (known as Q fever), which is a globally zoonotic disease.^[15]^ Populations who live in rural areas or had a history of animal exposure are at the high-risk of Q fever.^[16,17]^ However, due to the nonspecific and variable manifestations with only less than a half presented as asymptomatic, Q fever is difficult to diagnose.^[18,19]^ Most acute Q fever could be self-limited fever, while 4% to 5% of them could develop into chronic Q fever, which required long-term treatment with a risk of relapse.^[20,21]^ At present, doxycycline is the preferred treatment for children and adults with acute Q fever.^[22]^ For those who are intolerant, levofloxacin and cotrimoxazole could be utilized as alternative treatment.^[23]^

The reports of PDAP caused by *C. burnetii* are relatively rare. Chang et al^[9]^ initially demonstrated that *C. burnetii* infection could be manifested as peritonitis in a patient with type 2 diabetes mellitus who complained of fever and chills for 20 days. After administration of the levofloxacin for 15 days, the patient’s symptoms were swiftly resolved. Yilmaz et al^[10]^ first reported that *C. burnetii* could cause PDAP in a patient who had fever, headache, anemia, elevated blood sedimentation and CRP without thrombocytopenia. After treatment with a combination of doxycycline, ciprofloxacin, and rifampicin, the patient’s symptoms improved. Although patient in this study had mild anemia with thrombocytopenia, the thrombocytopenia might be attributable to long-term PD. In addition, this patient had no symptoms of fever, which made it difficult to suspect the diagnosis of *C. burnetii* infection. Moreover, as coronavirus disease 2019 was diagnosed during the standard treatment of broad-spectrum antibiotics, the increased WBC count during this period could not be attributed to the ineffective treatment or novel coronavirus.

The treatment regimen for our patient prior to mNGS was determined based on the guidelines for peritoneal PDAP and the clinical experience at our hospital. As the patient was admitted with PDAP and negative culture results from PD fluid, empirical treatment with vancomycin and eticlopidine was initiated to cover both gram-positive and gram-negative bacteria. Given the persistence of PDAP symptoms, including turbid PD fluid and an elevated WBC count, ticlopidine was added to address potential vancomycin-resistant enterococci and ampicillin-resistant bacteria. Piperacillin was introduced when upper respiratory tract infection symptoms developed, and moxifloxacin was added to account for possible infections, such as *Mycoplasma* or *Mycobacterium tuberculosis*.

## 4. Conclusion

In conclusion, in patients with culture-negative PDAP and a history of animal exposure, clinicians should be aware of the possibility of *C. burnetii* infection, even in patients without symptoms of fever. Prompt mNGS testing should be conducted to determine the presence of *C. burnetii* to avoid delayed treatment.

## Author contributions

**Data curation:** Yue Zhong, Ze-Yu Cai.

**Conceptualization:** Jian Hao.

**Formal analysis:** Yue Zhong, Ze-Yu Cai, Jun-Rui Wang, Qi-Ge Qi, Jian Hao.

**Funding acquisition:** Jian Hao.

**Investigation:** Yue Zhong, Ze-Yu Cai, Jun-Rui Wang, Qi-Ge Qi, Jian Hao.

**Methodology:** Yue Zhong, Ze-Yu Cai, Jun-Rui Wang, Qi-Ge Qi, Jian Hao.

**Project administration:** Jian Hao.

**Resources:** Yue Zhong, Ze-Yu Cai, Jian Hao.

**Software:** Yue Zhong, Ze-Yu Cai.

**Supervision:** Jian Hao.

**Writing – original draft:** Yue Zhong, Ze-Yu Cai.

**Writing – review & editing:** Jun-Rui Wang, Qi-Ge Qi, Jian Hao.
